# Pulsatile lavage systems and their potential to penetrate soft tissue

**DOI:** 10.1007/s00068-022-02067-x

**Published:** 2022-09-13

**Authors:** Kevin Knappe, Andre Lunz, Matthias Bülhoff, Mareike Schonhoff, Tobias Renkawitz, Jan Philippe Kretzer, Sebastian Jaeger

**Affiliations:** 1 Department of Orthopedic Surgery, Heidelberg University, Heidelberg, Germany; 2 Laboratory of Biomechanics and Implant Research, Heidelberg University, Heidelberg, Germany

**Keywords:** Pulsatile lavage, Soft tissue, Traumatic wounds, Septic surgery, Impact pressure, Flow rate

## Abstract

**Background:**

In orthopedic and trauma surgery, pulsatile lavage systems are used to clean soft tissue. This may be necessary in septic surgeries or in case of contaminated wounds after trauma. Positive features such as reduction of bacterial contamination and removal of foreign particles are counterbalanced by negative aspects such as bacterial seeding in deeper tissue layers, damage to various tissues and even cases of air embolism.

**Purpose:**

The aim of this prospective experimental in vitro study was to compare impact pressure and flow rate in three different pulsatile lavage systems and to determine, whether these parameters alter their ability to reach deeper soft tissue layers.

**Methods:**

To test the penetration of soft tissue, the muscle tissue was flushed with contrast medium instead of saline fluid and afterwards scanned by computed tomography.

**Results:**

Impact pressure and flow rate showed significant differences between the different systems. There were no significant differences between the three devices in terms of total penetration volume, but there were significant differences in penetration depth.

**Conclusion:**

In this study, we found that higher impact pressure leads to deeper penetration and therefore bacteria are likely to be transferred to deeper tissue layers.

## Introduction

High-pressure pulsatile saline lavage systems are used in different procedures during a variety of surgeries. Cleaning cancellous bone prior to cementing in joint arthroplasty is a primary purpose [1–6]. The second main area of application for pulsatile lavage systems is cleaning soft tissues during surgical treatment of infections or in case of contaminated wounds after trauma [7, 8].

Cleaning wounds by pulsatile lavage is an effective procedure to reduce bacterial contamination of different surfaces [9]. It also is an essential step during surgical treatment of periprosthetic joint infections [10]. Wound irrigation with high-pressure (0.34 N/mm^2^) saline lavage results in a significant reduction of bacteria in various types of wounds [7, 8]. Even though there are many different irrigation solutions and devices that can be used in contaminated musculoskeletal wounds, they do not seem to have a superior clinical outcome when compared to pulsatile saline lavage only [11]. It could also be shown that pulsating jet lavage was more effective in reducing bacterial population, removal of necrotic tissue and foreign particles from the wounds compared to using bulb syringe alone [12, 13]. There even are data indicating that three liters of pulsatile lavage is as effective as doing the same procedure with nine liters by bulb syringe [14].

On the other hand, negative aspects have to be considered using pulsatile lavage in soft tissue. In an in vitro study in contaminated human tibial fractures, high-pressure pulsatile lavage resulted in significant damage to the bone and intramedullary bacterial seedings [15]. Also, cases of air embolisms during pulsed saline lavage of pelvic fractures exist [16–18]. Furthermore, air that is pushed into the muscle can cause perioperative complications [19]. Recent examinations showed that high-pressure pulsatile lavage propagates bacteria into soft tissue [20], but there is no data showing that depth of penetration and more bacteria in deeper soft tissue layers lead to higher infection rates. It has also been shown that pulsatile lavage of musculoskeletal wounds at a pressure of 0.14 N/mm^2^ can lead to irreversible tissue damage, resulting in myonecrosis and dystrophic calcification in a rat model [21]. Boyd et al. also proved that high-pressure pulsatile lavage causes soft tissue damage [22, 23].

Various pulsatile lavage systems are available, but there are few data on the different physical parameters and their impact on soft tissue.

The aim of this prospective experimental in vitro study was to compare impact pressure and flow rate in three different pulsatile lavage systems and their ability to reach deeper soft tissue layers.

## Materials and methods

### High-pressure pulsatile lavage systems

**Fig. 1:**
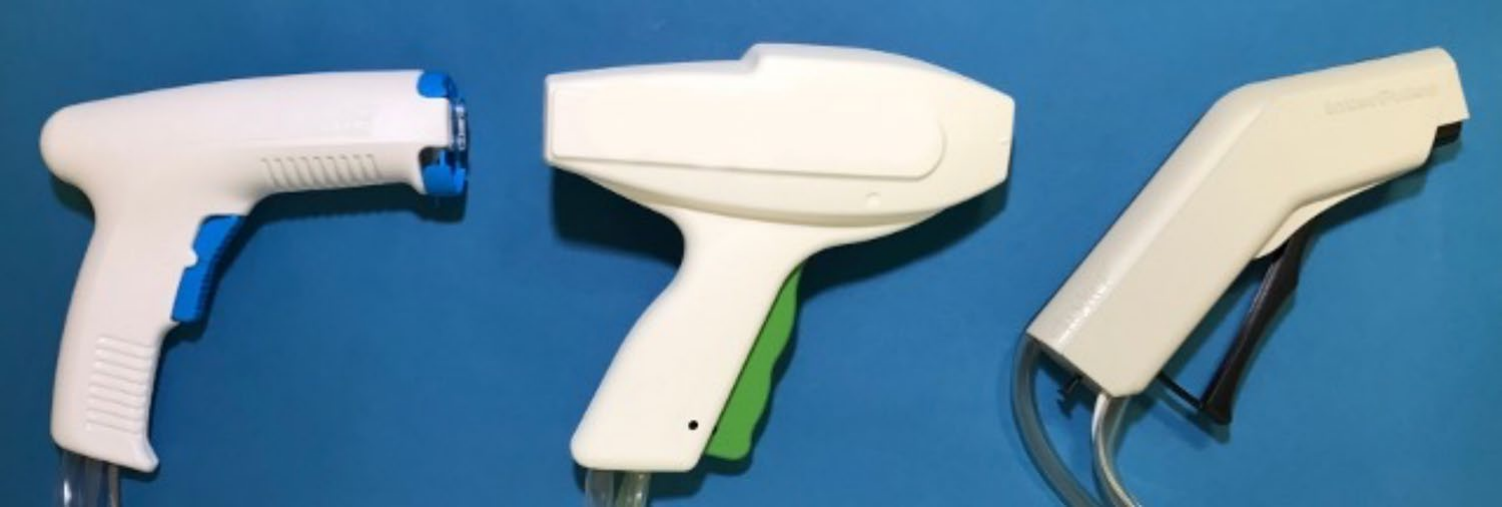
Handpieces of the three pulsatile lavage systems. (left) Group A: Pulsavac Plus, (middle) Group B: Vacuum Lavage, (right) Group C: InterPulse

**Fig. 2:**
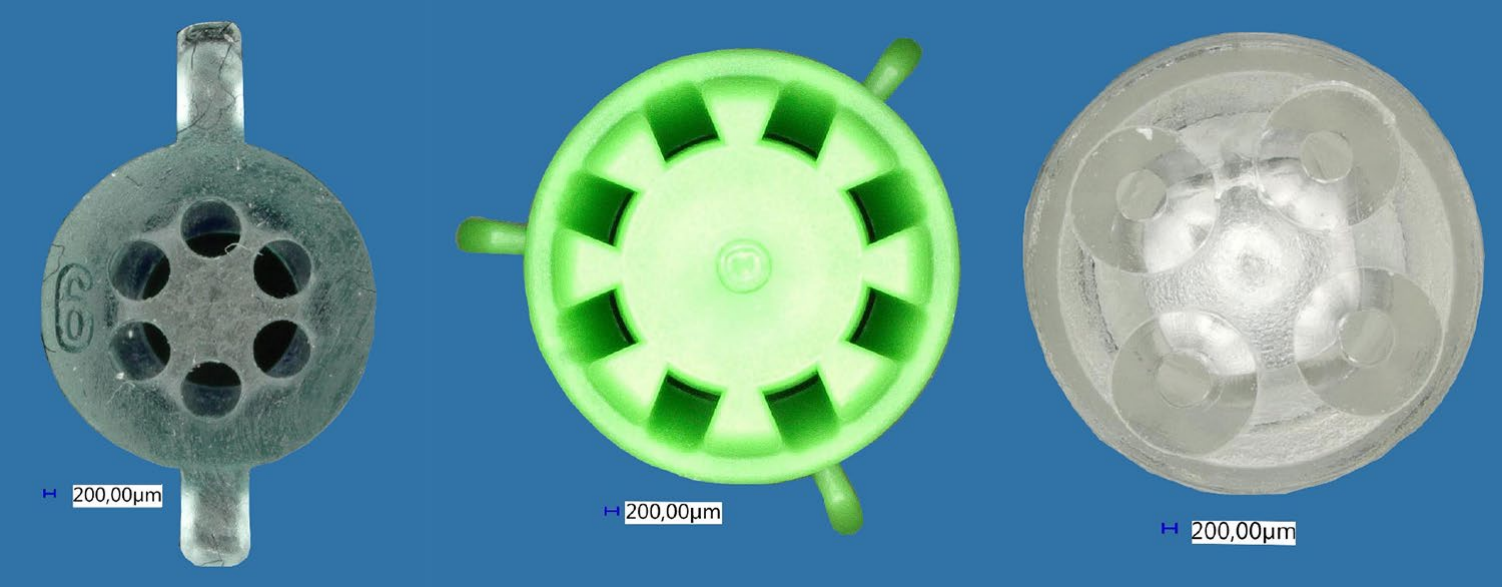
Front view of the three soft tissue tips and their nozzle orifices. (left) Group A: Pulsavac Plus, (middle) Group B: Vacuum Lavage, (right) Group C: InterPulse

In our experiments, we used three different devices from three different manufacturers that are used for pulsatile cleaning of soft tissue. All of them were single use devices. Two use battery power and one a new kind of vacuum driven system. For each lavage system the soft tissue tip that was recommended by the manufacturer was used. The systems were divided into three groups. Group A—Pulsavac Plus in its low-pressure mode and with the High Capacity Shower Spray Tip 00-5150-195-00 (Zimmer/Biomet, Warsaw, Indiana, USA). Group B—Vacuum lavage with its green colored soft tissue cleaning tip (development project, Heraeus Medical, Wehrheim, Germany). Group C—InterPulse with its Soft Tissue Tip 0210-012-0000 (Stryker, Kalamazoo, Michigan, USA) (Figs. 1 and 2). Saline fluid was used for all devices, according to the manufacturers’ recommendations.

### Cleaning parameters

First, the physical parameters were investigated by determining the impact pressure and flow rate of each device. For this purpose, an identical standardized set-up was established. To place the splash guard at a defined distance of 2 mm in front of a vertical force measuring plate, all systems were firmly fixed on an adjustable slide. The force was applied centrally on the force plate.

The splash guard specified the distance (2.0 mm + *X*) of the nozzles to the force measuring plate. The distance between the tip and the target was 41.0 mm in Group A, 18.0 mm in Group B and 19.0 mm in Group C, including 2 mm to let flushing medium run off. Nozzle orifices were measured too. The biggest outlet was shown by the green tip of Group B with 5.6 mm^2^, followed by the soft tissue tip of Group A with 5.1 mm^2^ and Group C with the smallest nozzle opening of 1.8 mm^2^ (Fig. 2).

Impact pressure was calculated by dividing the force and the area of the nozzle orifice Fig. 2). The nozzle openings of the three lavage systems investigated were measured using a calibrated digital microscope (Digital Microscope VHX-500 by Keyence, Osaka, Japan). Then, the areas were marked and calculated with ImageJ [24]. The maximum impact pressure was calculated during an already established 30 min test [25]. Maximum impact pressure was evaluated at 0.5, 5, 10, 15, 20, 25 and 30 min. To determine a mean maximum, a time interval of 60 s was used for each of the time markers named above. For example, the mean maximum was calculated from 9:30 to 10:30 (minutes: seconds) for the 10-min mark.

During the first 60 s of the experiment, the flow rate was measured.

### Soft tissue penetration

**Fig. 3:**
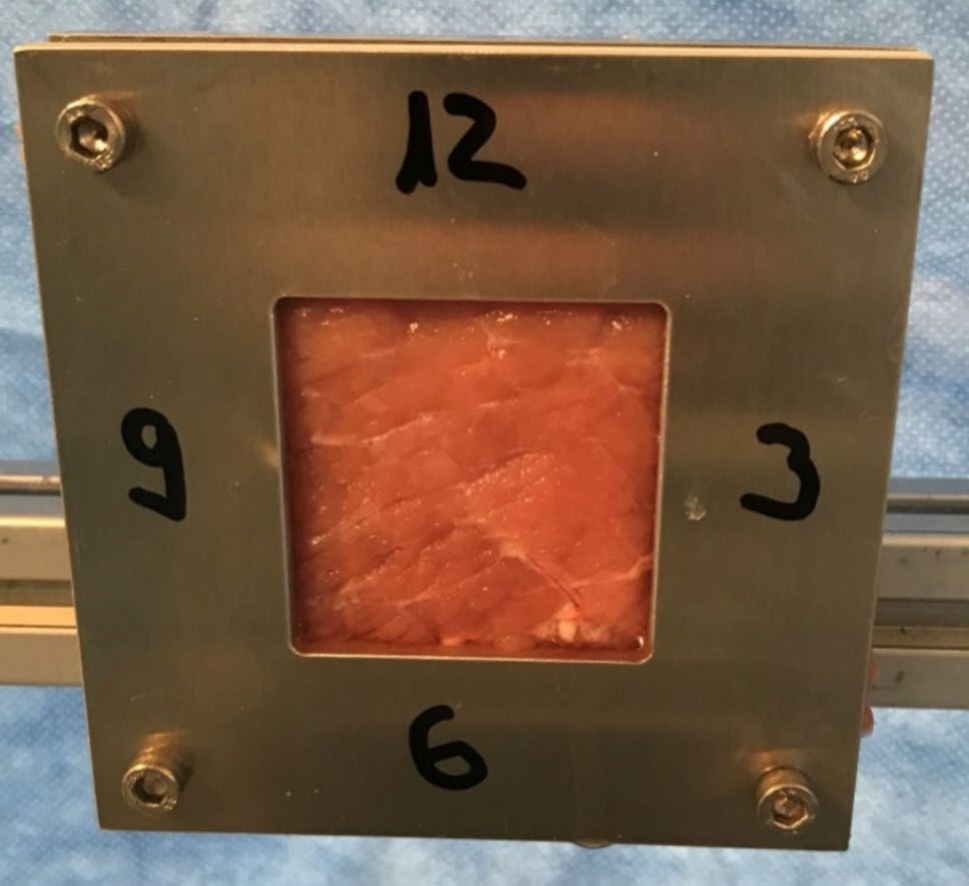
Pork flesh (5 × 5 cm) in holding device

Next, we investigated the effects of the three different systems when applied to soft tissue. Seven specimens were used in each group. To simulate human muscle, fresh industrial produced pork flesh was used, which was already sliced, and it was put into a customized holding device. The opening area of the holding device was 50 × 50 mm and positioned vertical to the ground so the flushing medium could easily run off (Fig. 3). Each of the 21 specimens was flushed with 500 ml of contrast medium (Ultravist-370, Bayer Vital GmbH, Leverkusen, Germany) that is usually used in Computed tomography (CT) diagnostics as an intravenous contrast medium. The distance to the surface was determined by the different splashing shields. During rinsing, the tip was constantly moved over the specimen with soft contact of the splashing shield to the ground, using the borders of the opening area as a guide to the splashing shield, so all of the contrast medium hit the specimens. The holding device was then removed and the edited tissue was cut out with a scalpel measuring 50 × 50 mm. Finally, the flesh was dabbed to take away a possible supernatant.

To evaluate the volume and the area of the contrast medium that penetrated the flesh, a CT scan (SOMATOM Emotion, Siemens Healthcare GmbH, Erlangen, Germany) with a slice thickness of 0.75 mm of all specimens was performed. To calculate the volume the programs ITK-SNAP [26] and Geomagic Studio Program (Raindrop Geomagic, NC, USA) were used. The penetration depth was calculated dividing the volume through the area. To find out significant differences between the groups, tests with ANOVA and a post hoc test Bonferroni were run by the program SPSS.

### Statistic

The data were evaluated descriptively using the arithmetic mean, standard deviation, minimum and maximum. Prior to the analysis, normal distribution of the data was evaluated using a Shapiro–Wilk test. Homogeneity of variance was verified using the Levene test. The requirements for the use of the ANOVA test were met and we conducted a one-way ANOVA to assess impact pressure, flow rate, penetration volume and penetration depth. The differences between the groups were evaluated using a Bonferroni test as post hoc analysis. The data were analyzed using SPSS 27 (IBM, Armonk, New York, USA) with a level of significance of *p* < 0.05.

## Results

### Impact pressure

Measuring the impact pressure, ANOVA showed significant differences of the investigated systems for all seven measurement times (ANOVA at 0.5 min: *F*(2,9) = 399.1, *p* < 0.001; at 5 min: *F*(2,9) = 550.5, *p* < 0.001; at 10 min: *F*(2,9) = 284.3, *p* < 0.001; at 15 min: *F*(2,9) = 350.3, *p* < 0.001; at 20 min: *F*(2,9) = 276.8, *p* < 0.001; at 25 min: *F*(2,9) = 280.1, *p* < 0.001; at 30 min: *F*(2,9) = 591.4, *p* < 0.001).

**Fig. 4:**
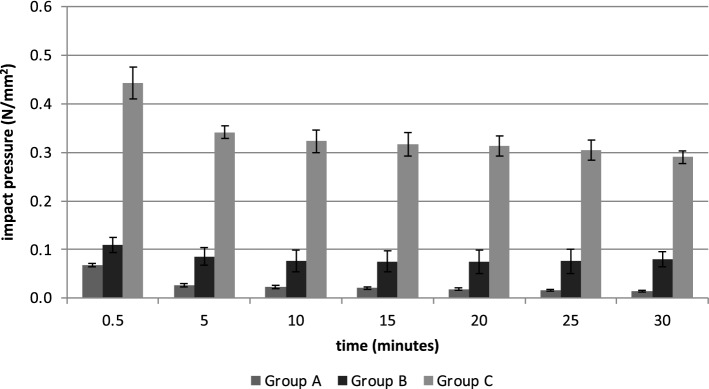
Impact pressure of high-pressure pulsatile lavage systems over time in a 30-min run test set-up

The maximum impact pressure in Group A was 0.07 ± 0.004 N/mm^2^ in the beginning and 0.01 ± 0.001 N/mm^2^ at the end. Group B started the test with 0.11 ± 0.02 N/mm^2^ and ended with 0.08 ± 0.02 N/mm^2^. Group C showed a maximum impact pressure of 0.44 ± 0.03 N/mm^2^ and ended with 0.29 ± 0.01 N/mm^2^ after 30 min. The results are summarized in Fig. 4.

### Flow rate

**Fig. 5:**
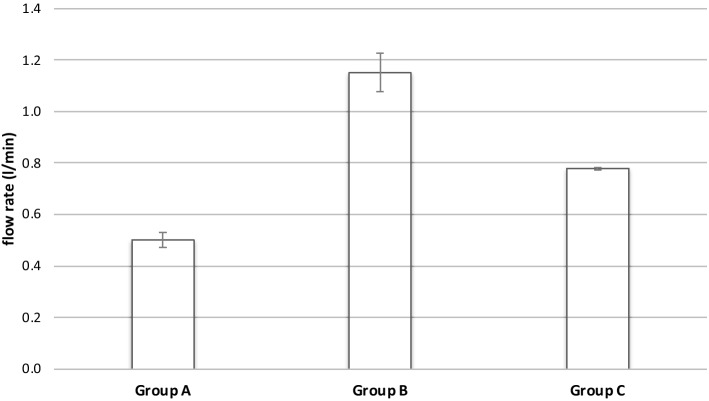
Flow rate of high-pressure pulsatile lavage systems in the first minute of their utilization

The flow rate was measured during the first minute of the test (Fig. 5). With 1.07–1.24 l/min Group B showed the highest flow rate (mean 1.15 ± 0.08 l/min), followed by Group C with 0.77–0.79 l/min (mean 0.78 ± 0.01 l/min) and Group A with 0.46–0.52 l/min (mean 0.50 ± 0.03 l/min). With a given normal distribution, ANOVA showed significant differences between groups (*F*(2,9) = 194.6, *p* < 0.001). A post hoc test revealed significant differences between each of the groups (Group A–B: *p* < 0.001; Group A–C: *p* < 0.001; Group B–C: *p* < 0.001) (Fig. 5).

### Effects on soft tissue

**Fig. 6:**
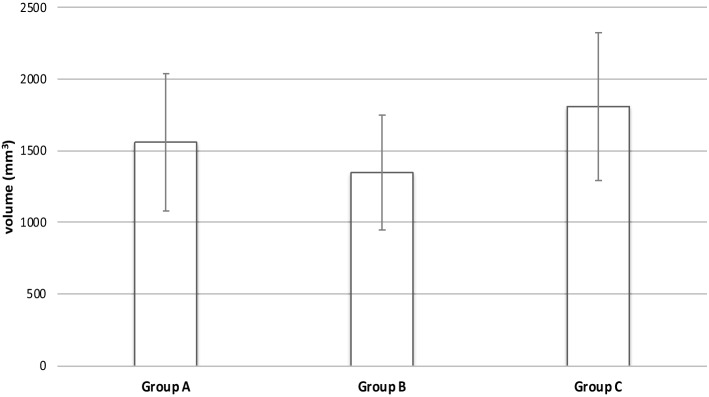
Volume of contrast medium that penetrated into soft tissue using different pulsatile lavage systems

The biggest mean volume of contrast medium in the treated specimens was shown by Group C (1808.9 ± 514.3 mm3; 766.6 to 2426.0 mm3). The second most volume was found in Group A with a mean volume of 1561.3 ± 478.7 mm^3^ (880.1–2296.0 mm^3^) followed by Group B with 1346.8 ± 403.1 mm^3^ (805.9–1845.0 mm^3^) (Fig. 6). All groups showed normal distribution. ANOVA revealed no significant differences (*F*(2,18) = 1.712, *p* < 0.209). The correlation coefficient of pressure and penetration volume in the 21 specimens was 0.33.

**Fig. 7:**
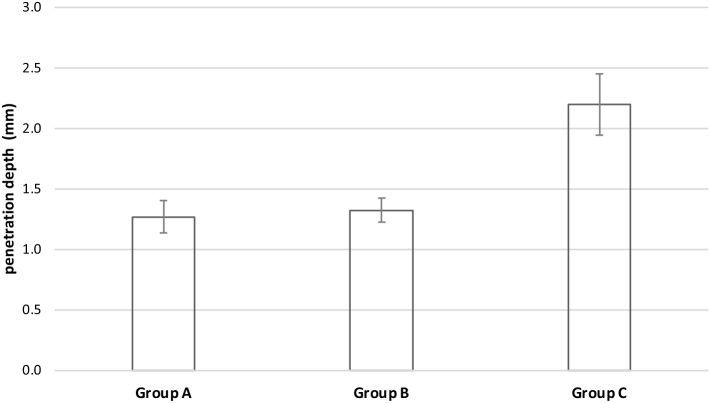
Penetration depth of different pulsatile lavage systems used in soft tissue

Looking at the penetration depth, Group C showed a mean depth of 2.2 ± 0.3 mm. The other two systems reached the same mean depth of 1.3 ± 0.1 mm (Fig. 7). ANOVA showed a significant difference between Group C and the less penetrating devices of Group A and B (*F*(2,18) = 62.203, *p* < 0.001). No significant differences between Groups A and B could be found (*p* = 1.0). The correlation coefficient of pressure and penetration depth in the 21 specimens was 0.93.

## Discussion

Pulsatile lavage can reduce bacterial contamination of different surfaces and different wounds [7–9]. Although it is more effective than bulb syringe alone [12, 13], research found that it can result in irreversible tissue damage [15, 21, 22].

Since there is only little data from different pulsatile lavage systems regarding their impact on soft tissue, the aim of this prospective experimental in vitro study was to determine whether impact pressure and flow rate of three different pulsatile lavage systems differ, and if this has any impact on penetration depth and penetration volumes into soft tissue.

Lavage system Group B had the highest mean flow rate with 1.15 l/min, which is significantly higher than in Group A (0.50 l/min) and Group C (0.78 l/min). However, high flow rates of flushing medium do not seem to have a relevant impact on penetrating volume as all 3 groups showed similar results. In both septic and trauma surgery, removing necrotic tissue and macroscopic contamination is key to primary wound healing. In addition to surgical debridement, wound irrigation is of outstanding importance [27, 28]. Higher flow rates are required to achieve higher flushing volumes in minimal time. Based on our experiments, we assume that high flow rates do not necessarily propagate more fluid into deeper layers.

The system with the longest distance to the soft tissue (Group A—41 mm) had the same penetration depth as the system with the shortest (Group B—18 mm). In both groups the area of the nozzle orifice was also similar large (Group A—5.1 mm^2^; Group B—5.6 mm^2^). But not only did they show the same penetration depth and similar areas of nozzle orifice, they also showed similar maximum impact pressure rates with 0.08 N/mm^2^ (Group A) and 0.11 N/mm^2^ (Group B). In contrast to that, Group C, which showed the highest penetration depth with 2.2 mm, operates with a nozzle orifice area of 1.8 mm^2^, which is less than 36% of the orifice area of Groups A and B. This small opening, from which the fluid emerges, produces an impact pressure four times higher compared to Group B and 5.5 times higher compared to Group A. Finally, the combination of a small opening with a high impact pressure results in the highest volume and significantly deeper penetration depth of the applied contrast medium. It remains subject to further research if higher impact pressure is solely caused by a smaller nozzle orifice. The different devices may have other characteristic that can lead to a higher impact pressure. The correlation coefficient of 0.93 of the measured impact pressure and the resulting penetration depth seems to prove the hypothesis that flushing medium hitting the specimens with higher pressure leads to a deeper penetration. According to Hassinger et al., it is more likely to propagate bacteria to deeper layers by high-pressure lavage than low pressure [20]. This also seems to prove the hypothesis of high pressure taking more bacteria to deeper layers. But, to the best of our knowledge, there are no data available whether bacteria that are propagated up to 2.2 mm into muscle tissue lead to more infections of deeper structures.

Latest reviews have doubted that high-pressure pulsatile lavage irrigation is able to increase the effectiveness of cleaning traumatic wounds in open fractures [29, 30]. Also, high pressure was not more effective than low-pressure irrigation in cleaning a contaminated wound in an experimental rat model [31]. Furthermore, the use of pulsed lavage and irrigation pressure of pulsed lavage do not seem to change the outcomes [32]. Acknowledging this, one option is to use a bulb syringe for small wounds, since this is an easy and cost-effective therapy. If larger wounds need to be treated, which requires higher irrigation volumes, the methodology of choice can be the fast, easy to handle and convenient pulsed lavage.

Using a laboratory set-up, we tested for penetration into muscle, since this is the most common type of soft tissue that has to be irrigated during septic and trauma surgery. Therefore, our experiments make no statement about other tissue types including subcutaneous fat, synovia, tendons or bone. However, the used set-up has a high level of standardization and the possibility to examine the results via a CT scan. Furthermore, we did not examine contamination with bacteria into deeper layers directly, we analyzed the penetration of contrast medium and made the assumption that bacteria and contamination are taken down by the flushing medium. If fluids take down bacteria into deeper layers stays uncertain. In addition, contrast medium is of course not a clinically used wound irrigation medium.

## Conclusion

According to the results of this experimental study, the use of pulsatile lavage systems with higher impact pressure in soft tissues could theoretically lead to deeper penetration of bacteria. Whether this effect is strong enough to be clinically relevant has to be part of further investigations.
